# Design of a Multi-Epitope Vaccine Based on *Fasciola gigantica* Cathepsin B and Evaluation of Immunological Responses in Mice

**DOI:** 10.3390/ijms26146971

**Published:** 2025-07-20

**Authors:** Supanan Chansap, Werachon Cheukamud, Thitikul Suthisintong, Pornanan Kueakhai, Narin Changklungmoa

**Affiliations:** Research Unit of Vaccine and Diagnosis of Parasitic Diseases, Faculty of Allied Health Sciences, Burapha University, Long-Hard Bangsaen Road, Saen Sook Sub-District, Mueang District, Chonburi 20131, Thailand; 62810076@go.buu.ac.th (S.C.); 61810046@go.buu.ac.th (W.C.); thitikul.su@go.buu.ac.th (T.S.); pornanan@go.buu.ac.th (P.K.)

**Keywords:** Cathepsin B, *Fasciola gigantica*, fasciolosis, immunoinformatic, multi-epitope, vaccines

## Abstract

*Fasciola gigantica* (*F. gigantica*) is a vital parasite that causes fasciolosis. Liver fluke infections affect livestock animals, and the *Fasciola* species (*Fasciola* spp.) vaccine has been tested for many types of these diseases. Currently, computer-based vaccine design represents an attractive alternative for constructing vaccines. Thus, this study aimed to design the epitopes of linear B-cells (BCL) and helper T lymphocytes (HTL) using an immunoinformatic approach and to investigate in silico and the mice’s immune response. A non-conserved host region, overlapping *F. gigantica* cathepsin B proteins (FgCatB), and the highest conserved residue percentages were the criteria used to construct epitopes. The GPGPG linker was used to link epitopes in the multi-epitope *Fasciola gigantica* cathepsin B (MeFgCatB) peptide. The MeFgCatB peptide has high antigenicity, non-allergenicity, non-toxicity, good solubility, and a high-quality structure. The molecular docking between the MeFgCatB peptide and Toll-like receptor 2 (TLR-2) was evaluated. The IgM, IgG1, and IgG2 levels were elevated in silico. In mice, the MeFgCatB peptide was synthesized and administered as an injection. The MeFgCatB-specific IgG1 and IgG2a levels were elevated after week 2, showing a predominance of IgG1. The rFgCatB1, rFgCatB2, and rFgCatB3 were detected using the MeFgCatB peptide-immunized sera. The MeFgCatB peptide-immunized sera were detected at approximately 28–34 kDa in the whole body. In addition, the MeFgCatB immunized sera can positively signal at the caecal epithelium in the NEJ, 4WKJ, and adult stages. In summary, the MeFgCatB peptide is able to induce mixed Th1/Th2 immune responses with Th2 dominating and to detect the native protein of *F. gigantica*. The MeFgCatB peptide should help against *F. gigantica* in future experiments.

## 1. Introduction

*Fasciola* species (*Fasciola* spp.) cause fasciolosis in ruminants such as cattle, sheep, and buffalo. *Fasciola gigantica* (*F. gigantica*) commonly infects tropical regions, while *Fasciola hepatica* (*F. hepatica*) is distributed in temperate regions worldwide. Moreover, there is an overlap in their distribution in Central Asia and East Africa [1,2]. Liver fluke infections affect the livestock economy in various ways, such as by decreasing dairy and meat production, leading to weight reductions, and producing barren animals [3,4,5]. According to the World Health Organization (WHO) report, the number of human fasciolosis cases is increasing worldwide, with an estimated 2.4 million people worldwide infected with *Fasciola* spp. in about 70 countries [6]. Currently, triclabendazole is used to treat infections caused by liver flukes; this drug is more effective than the available alternatives [7,8]. Members of the same drug group include phenoxyalkanes, benzimidazoles, salicylanilides, and sulphonamides [9]. Triclabendazole can treat ruminants infected with liver flukes in the immature and mature stages [10,11]. Nonetheless, it has been shown that liver flukes exhibit pharmaceutical resistance in numerous countries, including the Netherlands, Turkey, Chile, and Peru [12,13]. Therefore, vaccination represents an exciting alternative to prevent liver fluke infection. Vaccines are safer, more affordable, and more sustainable than drug treatments [14,15,16].

Liver flukes undergo multiple stages when entering the host organism. Each stage of liver flukes has different levels of protein expression. Liver flukes infect definitive hosts and excyst in the duodenum, which divides into two sub-stages, including pre-hepatic and hepatic stages. In a critical step in the pre-hepatic stage, the newly excysted juvenile (NEJ)’s liver flukes were excysted from the metacercariae and penetrated the intestinal mucosa, which damaged tissues in the duodenal submucosa. After that, it passes through to the serosa and enters the peritoneal cavity and matures into juvenile liver flukes. In the hepatic stage, the migrating juvenile liver flukes damage the liver parenchyma. The juvenile liver flukes develop into adult liver flukes in the bile duct [15,17]. Consequently, the immature and mature stages of the live fluke secreted proteins have been considered as possible vaccine proteins. A variety of proteins have been investigated for their potential as a fasciolosis vaccine, as determined by prior studies [18,19,20,21,22,23,24,25,26,27,28,29,30,31,32,33,34,35].

Cathepsins have been found to be a major protein, which is present in all stages of liver flukes [15,17]. Cathepsins play a crucial role in the disease’s survival in the host, including the parasite’s invasion, digestion, immune evasion, and excystment [14,36]. *F. gigantica* cathepsin B proteins (FgCatB) are an essential enzyme, which is highly expressed during all stages of liver fluke infections. The three isotypes of the FgCatB are *F. gigantica* cathepsin B1 (FgCatB1) protein, *F. gigantica* cathepsin B2 (FgCatB2) protein, and *F. gigantica* cathepsin B3 (FgCatB3) protein. FgCatB1 is expressed during all stages of the parasite [37]. FgCatB2 and FgCatB3 are highly expressed in metacercaria, NEJs, and juvenile parasites [38,39]. Therefore, designing a vaccine requires selecting proteins that facilitate the survival of the parasite in the host at all stages. Previously, studies have shown that FgCatB2 and FgCatB3 proteins have the capacity to enhance the immune system of the host and protect against parasitic infections [32,35].

The host’s immune system responds after the liver flukes enter the body. The immune responses to extracellular parasites involve both humoral immunity and cellular immunity [15]. The humoral immune response is a vital line of defense against parasitic infections because it produces antibodies. Moreover, the cellular immune response uses dendritic cells, macrophages, and Langerhans cells, which are examples of antigen presenting cells (APC) that are able to recognize parasite antigens. On T helper 2 (Th2) cells, APC delivers antigens through the major histocompatibility complex (MHC) class II. Th 2 cells secrete cytokines that enhance smooth muscle contraction, stimulate effector cells, encourage B-cells’ production of immunoglobulin, increase mucus production by the goblet cells, and increase epithelial cell permeability [40]. Furthermore, one of the main mechanisms of the humoral immune response to extracellular parasites is antibody-dependent cellular cytotoxicity, or ADCC. The ADCC process is initiated by a particular antibody that binds to a parasite antigen and activates effector cells, especially natural killer (NK) cells. The parasite antigen is destroyed by the ADCC mechanism, which results in degranulation [41].

Peptide or epitope vaccines represent a new technology in vaccine production. These vaccines use the parts of an antigen that can directly stimulate the immune responses [42,43]. Therefore, immunoinformatic tools are used to predict the epitopes of antigens with high antigenicity [44,45]. The mechanisms whereby antigens recognize immune cells are as follows: B-cells and T-lymphocytes (T-cells) recognize antigens via B-cell receptors (BCRs) and T-cell receptors (TCRs). T-cells present antigens to B-cells, which can produce antibodies. In addition, B-cells can recognize free antigens and produce antibodies [46,47]. In previous studies, the peptide vaccines were determined to be efficient against parasites, bacteria, and viruses in in vivo and in vitro testing, including *S. mansoni* [48], *Ascaris suum* (*A. suum*) [49], *Trichuris trichiura* (*T. trichiura*) [50], *Trichinella spiralis* (*T. spiralis*) [51], *F. gigantica* [52], *F. hepatica* [53], *Leishmania infantum* (*L. infantum*) [54], *Helicobactor pylori* (*H. pylori*) [55], severe acute reparatory syndrome coronavirus 2 [56], and influenza N2 neuraminidase [57]. In addition, in silico immune simulations are used to predict host immunity in the present [58]. Consequently, this study aimed to design the multi-epitope *Fasciola gigantica* cathepsin B (MeFgCatB) peptide, employing immunogenic epitopes predicted to be specific to helper T lymphocytes (HTL) and linear B-cells (BCL), and to determine the immune response levels in in silico and mice experiments.

## 2. Results

### 2.1. The Primary Analysis of Amino Acid Sequence Retrieval

For the FgCatB sequences, the PepCalc server, Vaxijen version 2.0, AllerTop version 2.0, and Expasy were used to predict their physicochemical characteristics, antigenicity, allergenicity, and solubility. The molecular weight (MW) and grand average of hydropathicity (GRAVY) scores ranged from 27.625 to 28.842 kilodaltons (kDa) and −0.559 to −0.419, respectively. The antigenicity ranged from 0.4678 to 0.5909. Furthermore, FgCatB2 and FgCatB3 were non-allergenic. FgCatB1 and FgCatB3 have good solubility (Table 1). An immunoinformatic approach was used to predict the mature FgCatB sequence. The properties of the BCL and HTL epitopes were validated. Molecular docking was used to dock the MeFgCatB peptide to Toll-like receptor 2 (TLR-2). Furthermore, the in silico immunological simulation identified the MeFgCatB peptide. Ultimately, the MeFgCatB peptide was synthesized and administered to mice, and the location and immune response were ascertained.

### 2.2. BCL Epitopes Prediction

**Figure 1:**
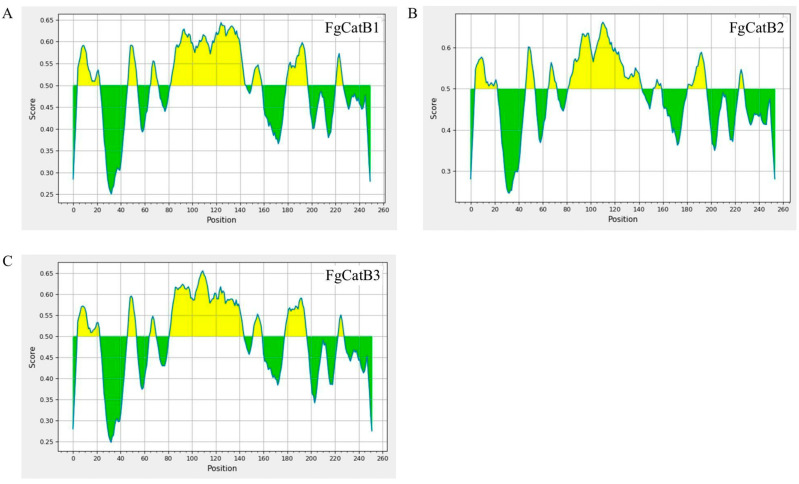
BCL epitopes of the *F. gigantica* sequences. (**A**) FgCatB1, (**B**) FgCatB2, and (**C**) FgCatB3 are linear B-cell epitopes. The yellow highlighted regions above the threshold line are BCL epitopes. The green highlighted regions below the threshold line are non-BCL epitopes. The cut-off score is 0.5 (where the Y and X axes represent the scores and positions of the residues of the sequence, respectively).

The BCL epitopes with scores higher than 0.5 were selected. In total, FgCatB1, FgCatB2, and FgCatB3 had 3, 2, and 2 epitopes, respectively. The antigenicity of the FgCatB1, FgCatB2, and FgCatB3 ranged from 0.9741 to 3.3484 and 1.7296 to 2.0161 and 0.7660 to 0.8798, respectively. All epitopes were non-allergenic (Table 2 and Figure 1).

### 2.3. HTL Epitopes Prediction

The FgCatB sequences were predicted to be 15 amino acids (aa) of the MHC II epitopes according to the Immune Epitope Database (IEDB) server. Antigenicity and allergenicity were used to select the HTL epitopes. In total, FgCatB1, FgCatB2, and FgCatB3 had 7, 7, and 5 epitopes, respectively. In addition, the antigenic scores of the FgCatB1, FgCatB2, and FgCatB3 ranged from 0.5161 to 1.6390, 0.6180 to 1.6318, and 0.6327 to 1.4574 (Table 3).

### 2.4. The Novel BCL and HTL Epitopes’ Selection and Construction

**Figure 2:**
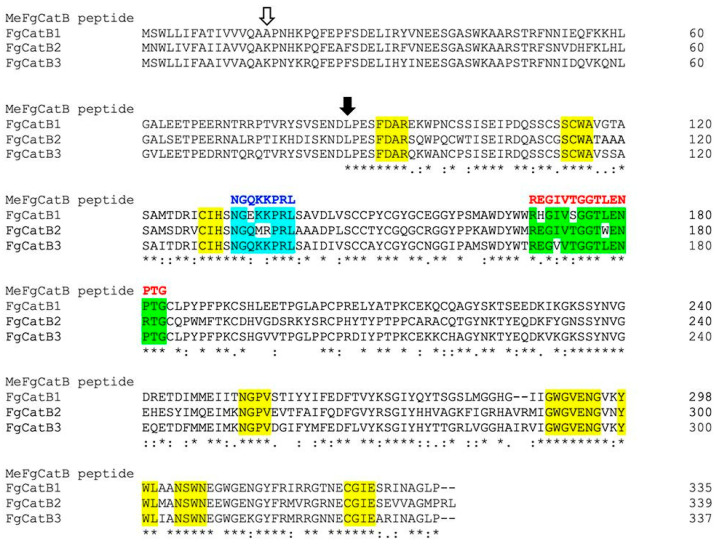
The BCL and HTL epitope sequences were selected and constructed. The mature protein is indicated by a solid arrow, while the pro-region is indicated by an open arrow. The region of the conserved host is indicated by a yellow label. The group of overlapped BCL and HTL epitopes is denoted by the cyan and green labels, respectively. The novel HTL and BCL epitope sequences are denoted by red and blue letters, respectively. The conserved residue locations of the FgCatBs are shown by an asterix (*), and the conservation across amino acid groups of similar FgCatBs features is indicated by a colon (:). The conservation between amino acid groups of the weakly comparable FgCatBs characteristics is indicated by the period (.).

The host’s cathepsin B proteins were in alignment with the selected BCL and HTL epitopes. Three criteria were used to choose the epitopes: the highest percentage of conserved residues, overlapping FgCatB sequences, and the non-conserved regions of the host. The overlapping groups of the selected BCL and HTL epitopes were groups 1 and 1 (cyan and green labels, respectively). The percentages of conserved residues of the BCL 1 (B1) and HTL 1 (T1) epitope groups were 62.50 and 73.33, respectively (Table 4). In Figure 2, the constructed novel BCL and HTL epitopes, shown in blue and red letters, were created using amino acids that were similar or identical to those of the overlapping groups of the selected BCL and HTL epitopes.

### 2.5. Design of the MeFgCatB Peptide

**Figure 3:**
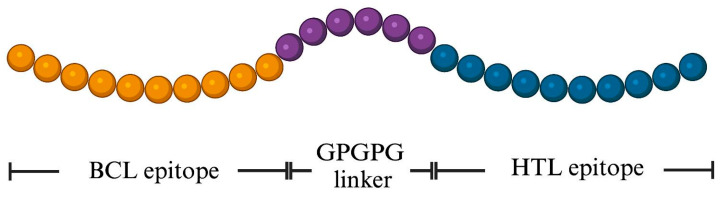
Schematic representation of the final MeFgCatB peptide. The length of the peptide is 28 aa. The yellow, violet, and blue colors represent the novel BCL epitope, the GPGPG linker, and the novel HTL epitope.

**Figure 4:**
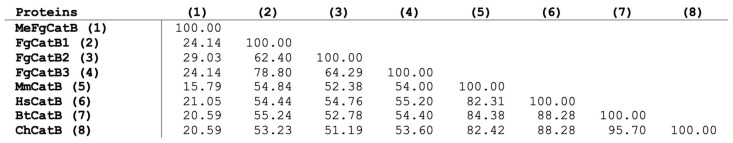
MeFgCatB peptide sequence identity matrix. Sequence identity matrix showing pairwise identity (0–100%) between the MeFgCatB peptide sequence and mature cathepsin B sequences from both the host and *F. gigantica*. MeFgCatB, multi-epitope-based *F. gigantica* cathepsin B; FgCatB1, *F. gigantica* cathepsin B1; FgCatB2, *F. gigantica* cathepsin B2; FgCatB3, *F. gigantica* cathepsin B3; MmCatB, *Mus musculus* cathepsin B; HsCatB, *Homo sapiens* cathepsin B; BtCatB, *Bos taurus* cathepsin B; ChCatB, *Capra hircus* cathepsin B.

The novel BCL and HTL sequences were used in the design of the MeFgCatB peptide. The GPGPG (glycine-rich) was employed to join the novel BCL and HTL epitopes, enhancing both immunogenic potential and peptide solubility. The final MeFgCatB peptide sequence contains 28 aa. The schematic design of the MeFgCatB peptide is shown in Figure 3. In addition, the sequence was aligned with the host’s cathepsin B and mature FgCatB sequences using the Clustal Omega v 1.2.4 servers. The identity matrix percentage ranged from 15.79 to 21.05% of the host’s cathepsin B and from 24.14 to 29.03% of the FgCatB (Figure 4). 

### 2.6. Predicting Solubility, Toxicity, Antigenicity, Allergenicity, and Physicochemical Characteristics

The physicochemical characteristics of the MeFgCatB peptide were assessed. A kDa of epitope was the MW. A value of 9.99 was the theoretical isoelectric point value (pI). A total of two negatively and four positively charged residues were found. A formula of C118H199N39O39 was used. The MeFgCatB peptide was found to have a half-life of 1.4 h in human reticulocytes, 3 min in yeast, and less than 10 h in *Escherichia coli* (*E. coli*). According to the instability index (II) of −15.93 (value < 40), the protein was determined to be stable. GRAVY and the aliphatic index were −1.061 and 52.14, respectively. The antigenic score of the peptide MeFgCatB was 0.5138. Additionally, MeFgCatB exhibited good solubility and was non-toxic and non-allergen.

### 2.7. Prediction, Refinement, and Validation of the MeFgCatB Peptide Tertiary Structure

**Figure 5:**
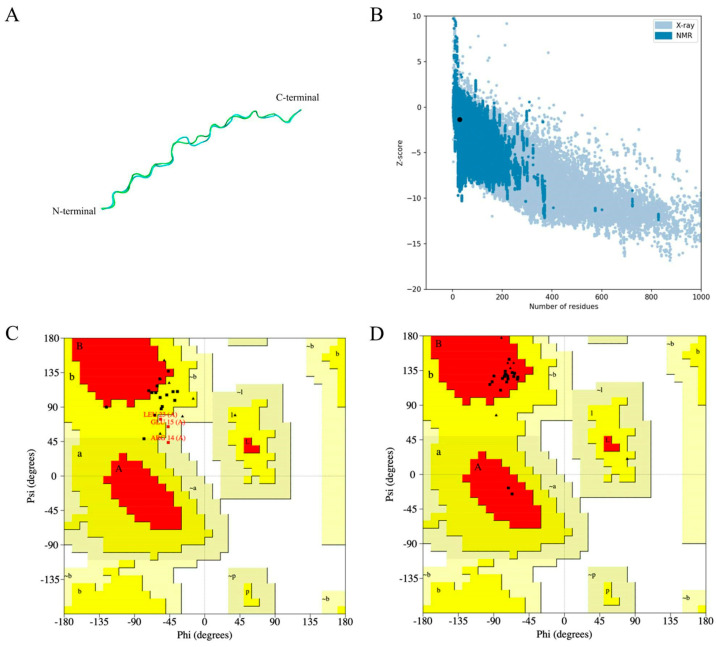
Prediction, refinement, and validation of the MeFgCatB peptide tertiary structure. (**A**) The MeFgCatB peptide’s tertiary structural alignment was indicated in the refined (cyan) and unrefined (green) models. (**B**) The Z-score (black dot) represents the model’s overall quality. Distinct structural groups have distinct colors (dark blue and light blue, respectively) depending on the source (X-ray, NMR). The calculated Z-score was −1.37. (**C**) Ramachandran plot of the unrefined model shows 26.7% in the most favored region, 53.3% in the additional allowed region, and 31% in the generously allowed region. (**D**) The refined model’s Ramachandran plot displays 100% in the most favored region. Red, dark yellow, and light-yellow colors represent the most favored region, the additional allowed region, and the generously allowed region, respectively.

The 3D structure was predicted using the AlphaFold2 server. The Ramachandran plot of the MeFgCatB peptide’s tertiary structure values display 26.7% in the most favored region, 53.3% in the additional allowed region, and 31% in generously allowed region. The MeFgCatB peptide model was refined using the GalaxyRefine server. The most favored region of the refined MeFgCatB peptide model’s Ramachandran plot displayed a score of 100%. In addition, the refined model was predicted to have a Z-score value of −1.37. These outcomes indicated their high-quality structure (Figure 5).

### 2.8. Predicting Discontinuous and Continuous B-Cell Epitopes

**Figure 6:**
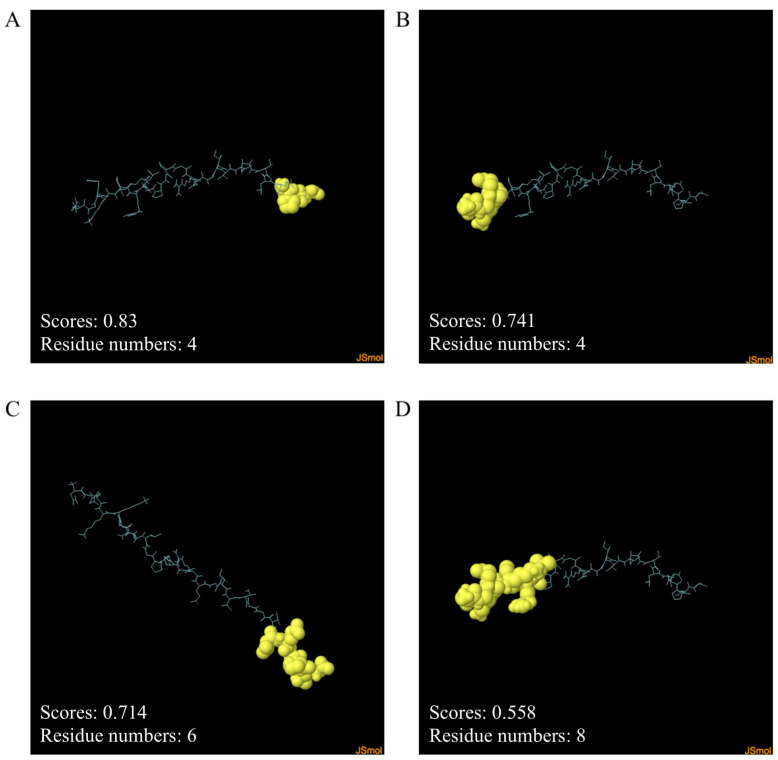
Predicting discontinuous and continuous B-cell epitopes. The discontinuous B-cell epitopes are shown in (**A**,**B**). The continuous B-cell epitopes are shown in (**C**,**D**). Cyan and yellow colors represent the epitope residues and the rest of the sequence, respectively.

The ElliPro server was used to predict the continuous and discontinuous B-cell epitopes. The scores for the two discontinuous B-cell epitopes were 0.83 and 0.741. The scores for the two continuous B-cell epitopes were 0.714 and 0.558, respectively. The results are shown in Figure 6 and Table 5.

### 2.9. MeFgCatB Peptide with TLR-2 Molecular Docking and Molecular Dynamic (MD) Simulation

**Figure 7:**
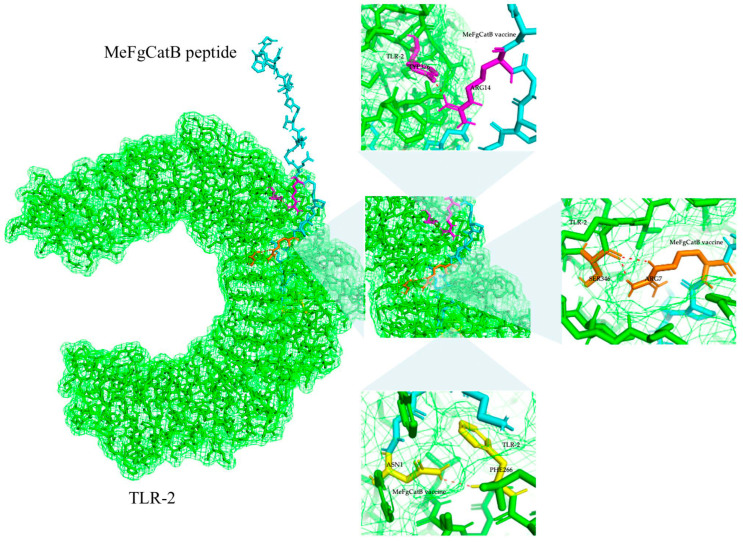
MeFgCatB peptide with TLR-2 molecular docking. A green stick and surface were used to identify the TLR-2 receptor. The molecular interaction was marked with red dotted lines. Magenta, orange, and yellow colors indicate ASN1-PHE266, ARG14-TYP376, and ARG7-SER346 residue interaction, respectively. The rest of the MeFgCatB peptide sequence indicates a cyan color.

The ClusPro2.0 server was utilized to assess the binding interaction between TLR-2 and the MeFgCatB peptide. There were 20 clusters in total. The molecular docking model was selected based on the fact that it had the lowest energy score. The lowest energy score shows the highest binding affinity between the receptor and the ligand, and it had a value of −896.3 kJ/mol. The molecular interactions between the MeFgCatB peptide model and TLR-2 residues were ASN1-PHE266, ARG14-TYP376, and ARG7-SER346 (Figure 7).

**Figure 8:**
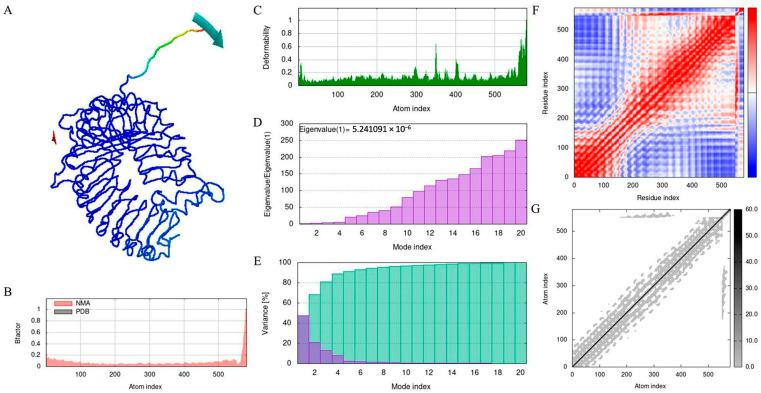
MD simulation analysis: (**A**) The MeFgCatB peptide–TLR-2 complex. NMA areas represent low mobility (red arrow) to high mobility (blue arrow). (**B**) The B-factor is the RMS and regional mobility score from 0 (lowest) to 1 (highest). (**C**) The deformability graph of the individual residues in the complex with lower distortion. (**D**) The eigenvalue is the motion stiffness score as 5.241091 × 10^−6^. (**E**) The eigenvalues, which show the individual (purple) and cumulative (green) variance, have a correlation that is inverse to the variance. (**F**) Correlated (red), uncorrelated (blue), and anti-correlated (white) motions are indicated by the covariance, which is the interaction between pairs of residues. (**G**) The elastic network model is a connection between two atoms. The grey indicates a higher protein stiffness in regions.

The MD simulation of the MeFgCatB peptide–TLR-2 complex was assessed using the iMODS server. In Figure 8, the mobility of the complex model was analyzed using normal-mode analysis (NMA). Red and blue represent the regions with the highest and lowest motion, respectively. The MeFgCatB peptide–TLR-2 complex has mobility through the B-factor and deformability. RMS, or root mean square, is the B-factor. The relationship between the complexes in the NMA and PDB regions is displayed in the B-factor graph. The deformability indicates high peaks corresponding to regions in protein deformability. The deformability graph shows a fairly high peak, indicating the deformation in that region. The eigenvalue indicates that the motion stiffness score was 5.241091 × 10^−6^. The variance graph shows inverse correlations between the individual (purple) and cumulative (green) variances. The covariance uses sequence alignments to calculate the coupling between pairs of positions in the sequence. In the covariance matrix, correlated, uncorrelated, and anti-correlated motion are represented by the colors red, white, and blue, respectively. Additionally, a range of protein complexes’ dynamics may be predicted using the elastic network model, which makes it possible to investigate a protein complex’s stiffness. Higher amounts of protein stiffness are seen in the gray areas. The MeFgCatB peptide–TLR-2 combination appears to be stable based on all the data.

### 2.10. In Silico Immune Response Simulation

**Figure 9:**
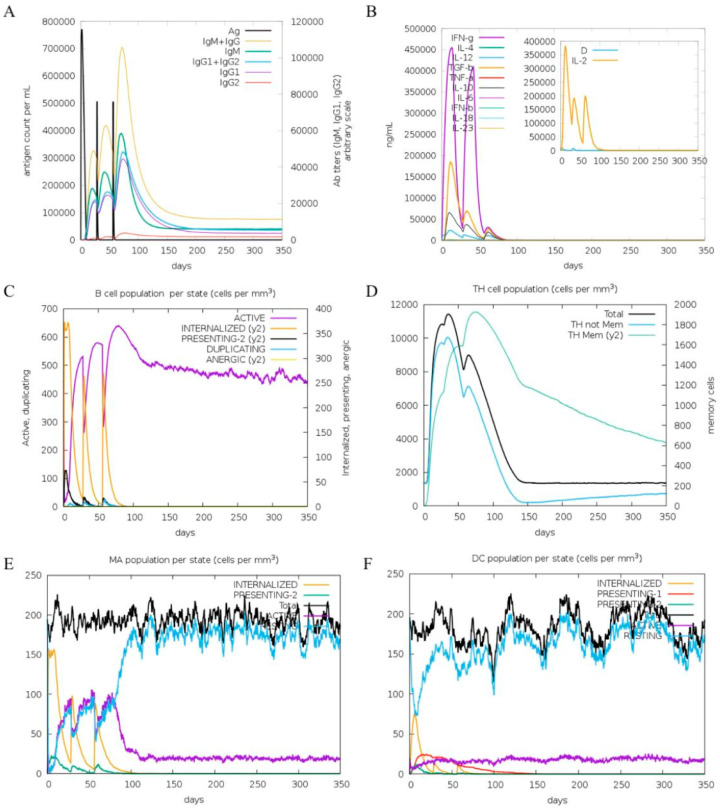
In silico immune response simulation: (**A**) antibody and antigen levels; (**B**) cytokine concentrations; (**C**) B-cell population by entity state; (**D**) T-cell population by entity state; (**E**) macrophage (MA) population per state; (**F**) dendritic cell (DC) population per state.

The C-immsim server was used for predicting the immunological simulation. Figure 9 displays the immunological responses that were activated following three dosages. At all doses, there was an increase in IgM, IgG1+IgG2, and IgM+IgG antibody levels (Figure 9A). The MeFgCatB peptide elevated interleukin-10 (IL-10), interleukin-12 (IL-12), transforming growth factor beta (TGF-β), and interferon gamma (IFN-γ) levels after immunization (Figure 9B). The active B-cell and HTL populations were shown in Figure 9C,D. In addition, the levels of active macrophages and dendritic cells were elevated, indicating effective antigen presentation (Figure 9E and Figure 9F, respectively).

### 2.11. The Levels of IgG1 and IgG2a

**Figure 10:**
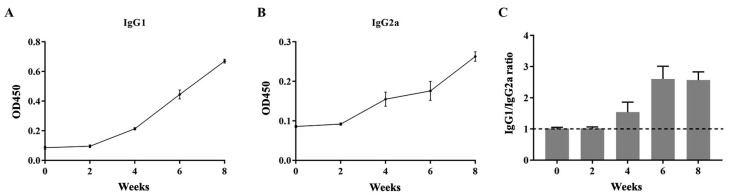
Levels of MeFgCatB-specific IgG1 and IgG2a antibodies: (**A**) the levels of MeFgCatB-specific IgG1 antibodies; (**B**) the levels of MeFgCatB-specific IgG2a antibodies; (**C**) calculated IgG1/IgG2a ratios for each mouse. The dashed line represents the IgG1/IgG2a ratio that equals 1.

**Figure 11:**
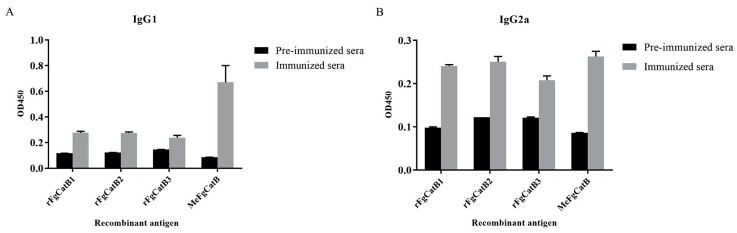
The MeFgCatB-specific IgG1 levels against rFgCatB1, rFgCatB2, rFgCatB3, and MeFgCatB (**A**), and the MeFgCatB-specific IgG2a levels against rFgCatB1, rFgCatB2, rFgCatB3, and MeFgCatB (**B**).

T helper 2 (Th2) and Th1 immune responses were denoted by the levels of IgG1 and IgG2a, respectively. Biweekly MeFgCatB peptide-immunized with Quil-A adjuvant sera and pre-immunized sera were used to measure the levels of IgG1 and IgG2a. The OD450 values of the MeFgCatB-specific IgG1 and IgG2a levels formed the background in pre-immunized sera. The levels of the MeFgCatB-specific IgG1 and IgG2a were highly increased after week 2, which were higher than pre-immunized mice sera (Figure 10A,B). The MeFgCatB peptide dominantly induced an IgG1 response. Consistent with this, the calculated IgG1/IgG2a ratios at each time point indicated a shift toward a Th2-dominant immune response after week 4 (Figure 10C). Sera collected at 8 weeks post-immunization showed significantly higher levels of MeFgCatB-specific IgG1 and IgG2a antibodies compared to pre-immunized sera. Additionally, MeFgCatB peptide-immunized sera exhibited elevated IgG1 and IgG2a antibody responses against the individual recombinant proteins rFgCatB1, rFgCatB2, and rFgCatB3, compared to pre-immunized sera (Figure 11).

### 2.12. Immunoblotting Analysis

**Figure 12:**
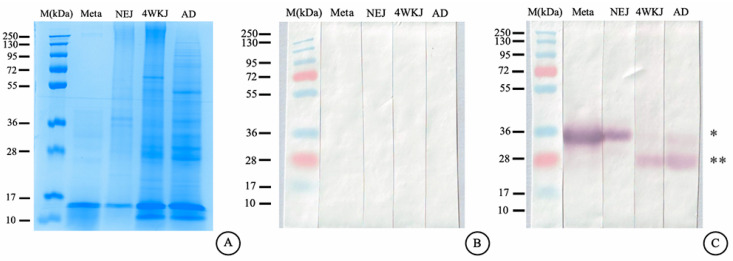
The Coomassie-stained SDS-PAGE of the WB’s *F. gigantica* and immunoblotting analysis. (**A**) Coomassie-stained SDS-PAGE showing protein profiles of whole-body extracts from metacercariae (Meta), newly excysted juveniles (NEJ), 4-week juveniles (4WKJ), and adults (AD). (**B**) The pre-immunized mice serum did not show a positive band in the WB’s *F.gigantica* in all stages (Meta lane–AD lane). (**C**) The MeFgCatB immunized mice serum detected a positive band in the WB’s *F.gigantica* in all stages (Meta lane–AD lane). M, marker lane; Meta, metacercariae; NEJ, newly excysted juvenile; 4WKJ, 4 weeks juvenile; AD, adult. Asterix (*) indicates a molecular weight of approximately 34 kDa. Double asterisks (**) indicate a molecular weight of approximately 28 kDa.

All band proteins of native whole-body (WB) F. gigantica were shown by the 12.5% SDS-PAGE with Coomassie blue stained (Figure 12A). The MeFgCatB immunized mice sera were used to investigate in immunoblotting analysis. Sera from pre-immunized mice did not show any positive bands in metacercariae, newly excysted juveniles (NEJ), 4-week-old juveniles (4WKJ), or adult stages (Figure 12B). Sera from MeFgCatB-immunized mice reacted with WB antigens from all developmental stages of F. gigantica, including metacercariae, newly excysted juveniles (NEJ), 4-week-old juveniles (4WKJ), and adults (AD). Sera from MeFgCatB-immunized mice detected a positive band at approximately 28–34 kDa in WB antigens of metacercariae. Similarly, a positive band at approximately 28–34 kDa was observed in WB antigens from the NEJ stage. Positive bands in the 4WKJ and adult stages were observed at approximately 28–34 kDa (Figure 12C).

### 2.13. Immunolocalization of the F. gigantica Tissue

**Figure 13:**
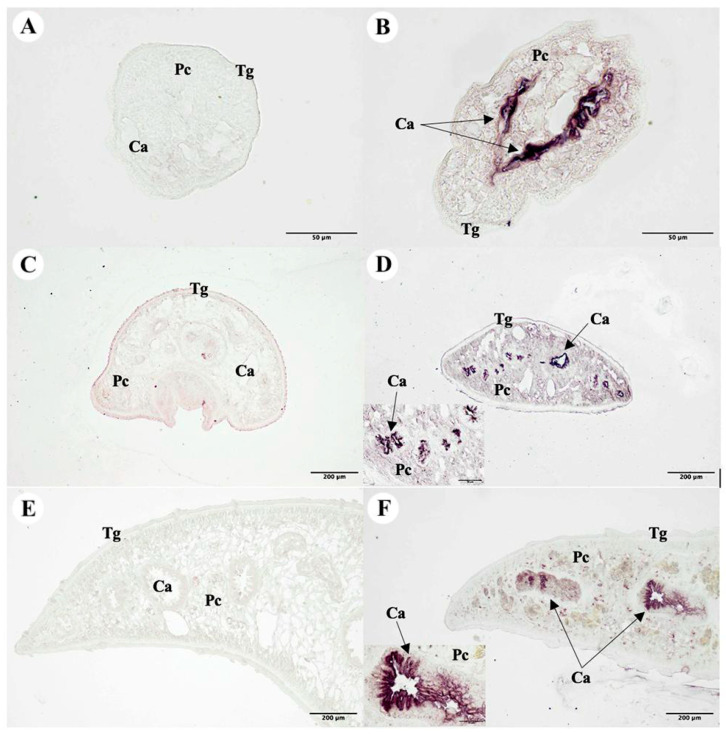
The MeFgCatB peptide-immunized sera localization. (**A**,**C**,**E**) Pre-immunized sera that were not stained in the NEJ, 4WKJ, and adult stages of the *F. gigantica* section were utilized as the negative control. (**B**,**D**,**F**) Purple staining in the cecal epithelium (Ca) of the NEJ, 4WKJ, and adult stages of *F. gigantica* tissue indicated the presence of MeFgCatB peptide-immunized sera. The parenchyma (Pc), vitelline gland (Vi), and tegumental cell (Tg) were not stained.

The immunolocalization approach was used to tissue sections of *F. gigantica* in the NEJ, 4WKJ, and adult stages. Pre-immunized sera that showed no positive signal in the *F. gigantica* section were employed as the negative control. Purple staining was used to detect the specific location of the MeFgCatB peptide-immunized sera in the cecal epithelial cells (Ca) of the NEJ, 4WKJ, and adult *F. gigantica*. Figure 13 shows that the parenchyma (Pc), vitelline gland (Vi), and tegumental cell (Tg) were not stained.

## 3. Discussion

Vaccines are crucial for both preventing diseases and enhancing the host’s immunity. The critical factors in vaccine production are low costs and long-term protection [59]. In fasciolosis, the recombinant proteins were used in tests, which aim to prevent *Fasciola* spp. infections [28,29,31,33,34,35]. Currently, immunoinformatic tools can predict specific B-cell and T-cell immune responses [47,60,61]. Many previous studies used peptide or epitope vaccines to prevent parasites, bacteria, and viruses in vivo and in vitro [48,49,50,51,52,53,54,55,56]. Therefore, in this study, the MeFgCatB peptide was designed by using immunoinformatic tools and the immune response was determined in in silico and mice experiments. The BCL and HTL epitopes from the mature FgCatB sequences were predicted using immunoinformatic methods. The selected BCL epitopes of FgCatB1, FgCatB2, and FgCatB3 had three, two, and two epitopes, respectively. The antigenic score ranged from 0.7660 to 3.3484. The selected HTL epitopes of FgCatB1, FgCatB2, and FgCatB3 had 7, 7, and 25 epitopes, respectively. The antigenic scores ranged from 0.5161 to 1.6390. The HTL epitopes are a specific sequence that Th cells recognize via APC [44]. Furthermore, the BCL epitope is a specific sequence that recognizes B-cell receptors or produces antibodies, activating the immune response of cells [60].

The mature FgCatB sequences of the host were aligned with the selected BCL and HTL epitopes throughout the epitope selection and construction process. The epitopes were selected from three criteria: the host’s non-conserved region, FgCatB sequence’s overlapping, and the highest percentage of conserved residue. Ancestral species that share particular areas or residues are known as conserved regions [62]. Only non-conserved host regions have been selected for this investigation. In addition, the constructed epitopes were created using overlapping FgCatB sequences and the highest-percentage conserved residue. Consequently, the novel BCL and HTL epitopes were a combined sequence of the FgCatB isotypes. The immune system probably targets the combined FgCatB isotype and recognizes this antigen, potentially providing protection against *F. gigantica* infection. In addition, the combined FgCatB isotype can probably protect other parasite species in a phenomenon referred to as cross-protection. Cross-protection is an important concept in the development of vaccines for infectious diseases [63,64]. The BCL and HTL epitopes contain a total of 8 and 15 amino acids, respectively. According to prior studies, the lengths of the BCL and HTL epitopes varied from 5 to 22 and 15 to 24 amino acids, respectively [65].

The novel BCL and HTL sequences were joined using a GPGPG linker. The linker sequence is a short amino sequence that cannot stimulate the immune system [66]. Furthermore, the linker was used to prevent the development of neo-epitopes, which is an important concern in vaccine construction [67,68]. GPGPG (glycine-rich) linkers were used to separate the novel BCL and HTL epitope sequences. Linkers play a critical role in facilitating humoral immune responses and can enhance the presentation of epitopes to helper T lymphocytes (HTLs) [69]. Furthermore, GPGPG linkers play a role in flexibility, protein folding, and increased solubility [70]. The protein identity of the vaccine candidate was used to evaluate the similarity between the MeFgCatB peptide, the host’s mature cathepsin B, and mature FgCatB sequences. According to the results, the MeFgCatB peptide was more similar to the FgCatB protein than the host’s cathepsin B protein. The similarity probably leads to cross-reactivity. Similar proteins in the host may be destroyed by the immune system’s reaction to a pathogen’s harmful antigens, ultimately resulting in autoimmune disease [71].

The results of our analyses show that the MW and pI of the MeFgCatB peptide were 2.79 kDa and 9.99, respectively. Its half-life was calculated to be less than 10 h in *E. coli*, 3 min in yeast, and 1.4 h in mammalian reticulocytes. The instability index (II) was −15.93, indicating that the protein was stable (II value < 40) [72]. The aliphatic index and GRAVY value were 52.14 and −1.061, respectively. The antigenicity was 0.5138, and the parasite antigenicity cut-off value is 0.5. Antigenicity refers to an antigen’s capacity to bind to or interact with the final products of the host’s immune system, including the production of cytokines, antibodies, and self-tolerance [73]. Additionally, the MeFgCatB peptide was also non-toxic, non-allergic, and had good solubility.

The tertiary structure was assessed using the AlphaFold2 server. The model with the highest pLDDT value was refined using the GalaxyRefine server. Ramachandran plots predict structural stereochemical properties and visualize energetically allowed and forbidden regions [74]. The MeFgCatB peptide model, with a value of 100%, is displayed in the most favored region of the Ramachandran plot. The tertiary structure’s quality was indicated by the z-score, which was used to validate the model [75,76]. The model was calculated to have a value of −1.37. Therefore, the model falls into the range of NMR-solved protein structures that indicate a high-quality model.

B lymphocytes are crucial in neutralizing antibody reactions to liver flukes in the early stages of the *F. gigantica* infection [77]. Discontinuous or continuous B-cell epitopes were predicted using the EliPro server. Two discontinuous B-cell epitopes were identified, and their scores were 0.83 and 0.741. With values of 0.558 and 0.714, two continuous B-cell epitopes were found. The ClusPro2.0 server was utilized to assess the molecular docking of the MeFgCatB peptide model in interactions with TLR-2. The surface of APC, which includes dendritic cells, monocytes, and macrophages, expresses TLR-2 [78]. The TLR-2 receptor plays an essential role in promoting humoral and cellular immune responses [79]. The immunomodulatory effect of TLR2 can also be directly applied to the humoral and cellular immune response [80]. Chain A of TLR-2 is responsible for the molecular recognition of numerous pathogens, including bacteria, viruses, fungi, and parasites [81]. Previous studies that investigated the interactions between fasciolosis vaccines employed the TLR-2 receptor [79,82,83]. The model with the highest binding affinity has the lowest energy score [84]. According to calculations, the lowest energy score was −896.3 kJ/mol. The molecular interactions of the MeFgCatB peptide model and the TLR-2 residue were ASN1-PHE266, ARG14-TYP376, and ARG7-SER346. In addition, an MD simulation of the MeFgCatB peptide model, the TLR-2 complex, was investigated using the iMODS server. The IMODS server was used to conduct a rapid molecular dynamic simulation study [85]. All results indicate the complex to be stable.

Furthermore, the MeFgCatB peptide was investigated regarding the immune response using the C-ImmSim server. Previous research involved evaluating peptide or epitope vaccines for parasites using the in silico immune simulation, with tests including *Schistosoma masoni* (*S. mansoni*) [86], *F. gigantica* [79,82], *F. hepatica* [84], *Entamoeba histolytica* (*E. histolytica*) [87], *Leishmania donovani* (*L. donovani*) [88,89], and *Naegleria fowleri* (*N. fowleri*) [90]. The peptide vaccines were found to elicit humoral and cellular immune responses [86,89]. The MeFgCatB peptide’s results showed that after the first dose, the levels of IgM and IgG antibodies increased. The active B-cell and HTL populations were elevated, indicating a long-lasting immune response and a stimulated cellular immune response, respectively [91]. IFN-γ, TGF-β, IL-10, and IL-12 were elevated. TGF-β and IL-10 were produced by M2 macrophages, leading to enhanced Th2 and T regulator (Treg) cell differentiation [40,92]. On the other hand, IL-12 is able to induce Th1 cell differentiation. However, the parasite’s survival mechanism inhibits the ability to stimulate immune cells. Consequently, the immune response fails to induce the development of antigen-specific Th1 and T helper 17 (Th17) cell-mediated immunity. IFN-γ was produced by Th1 cells, stimulating inducible Nitric oxide synthase (iNOS) expression and can promote parasite killing [92,93]. Increasing IL-12 and IFN-γ probably helps in the parasite clearance mechanism. In addition, the levels of active macrophages and dendritic cells were elevated. Therefore, the predicted MeFgCatB peptide was able to mimic the natural immunity induced by antigens [86]. According to immunoinformatic investigations and in silico immunological simulations, the MeFgCatB peptide could stimulate cellular immune responses by increasing important cytokines associated with helper T-cell activation, including IFN-γ, IL-10, and IL-12. Similar in silico approaches have previously been used to predict cytokine profiles in other vaccine studies, supporting the relevance of our simulation results [86,91,94,95].

The adjuvant is an important component that helps to stimulate the host’s immune response. The Quil-A adjuvant is an aqueous saponin derived from the bark of *Quillaja saponaria.* Aqueous saponin can induce a strong immune response in T-dependent and T-independent antigens [96]. Quil-A adjuvant is widely used in veterinary and human vaccine testing to induce a mixed Th1/Th2 immune response [97,98]. In this study, the MeFgCatB peptide with Quil-A adjuvant was investigated in the immune response levels. The IgG1 and IgG2a levels were increased after week 2 without any observed signs of toxicity or side effects in the immunized mice. The animals remained active and showed no clinical abnormalities. The use of Quil-A, an adjuvant with a well-established safety profile in veterinary vaccines, likely contributed to the absence of adverse effects [98]. This finding is consistent with previous studies describing the safety profile of Quil-A in veterinary applications [97]. IgG1/IgG2a ratios at each time point indicated a shift toward a Th2-dominant immune response, consistent with previous studies suggesting that a ratio greater than 1 is associated with a Th2-biased immune response. In contrast, a ratio below 0.5 is generally considered unrelated to Th2-type activation [99,100]. The increasing ratio after week 4 suggests a shift toward Th2-dominant immunity. This observation aligns with prior research, such as immunization with recombinant FhSAP2, where protection correlated with a strong IgG1 response, reflected in a high IgG1/IgG2a ratio [101]. Such Th2 responses are typically linked to mechanisms like antibody-mediated neutralization and ADCC [41,102]. In addition, MeFgCatB-specific IgG1 and IgG2a levels against rFgCatB1, rFgCatB2, and rFgCatB3 were significantly higher than those in pre-immunized sera, indicating that sera from MeFgCatB peptide-immunized mice can recognize each of the individual recombinant proteins, including rFgCatB1, rFgCatB2, and rFgCatB3. The pattern of immune response in mice immunization indicated a similar in silico immune simulation. In addition, this results in similar previous studies in rFgCatB2 and rFgCatB3 [35]. The results showed increased levels of IgG1 and IgG2a, indicating that the MeFgCatB peptide was capable of inducing both humoral and cellular immune responses. Therefore, the MeFgCatB peptide probably can replace the recombinant protein vaccine in the future. In previous studies, the peptide sequences have mimicked the viral spike of HIV-1 that can reduce morbidity and mortality via depression of viral replication [103]. In addition, the peptides have protein functions that are similar to the natural proteins [104].

The serum from mice immunized with the MeFgCatB peptide detected native cathepsin Bs in all stages of *F*. *gigantica*, including metacercariae, NEJ, 4WKJ, and adult stages. Western blot analysis revealed specific bands ranging from approximately 28 to 34 kDa in all stages. These findings are consistent with previous studies reporting detection of rFgCatB2 and rFgCatB3 proteins at similar molecular weights in the metacercariae and NEJ stages. These bands likely correspond to the non-glycosylated forms, approximately at 28 kDa, and glycosylated forms, approximately at 34 kDa, of cathepsin Bs [38,39]. Furthermore, the FgCatB1 gene is known to be expressed in all *F. gigantica* life stages [37], which supports the ability of the MeFgCatB peptide to detect all stages of the parasite, likely due to its specificity for the native FgCatB1 protein. In addition, the *F. gigantica* tissue was identified using sera immunized with the MeFgCatB peptide. Immunolocalization analysis further demonstrated that sera from immunized mice recognized antigens in the caecal epithelium of NEJ, 4WKJ, and adult stages of *F. gigantica*, a known site of cathepsin B expression [37]. These findings align with prior reports involving rFgCatB2 and rFgCatB3 and suggest that MeFgCatB may interfere with the parasite’s digestive functions [38,39]. Together, the Western blot and immunolocalization data indicate that immunization with the MeFgCatB peptide generates antibodies capable of recognizing native cathepsin B proteins across all stages of *F. gigantica*.

However, there are some limitations to consider, including peptides often being low immunogenic, meaning co-stimulators are required, peptides can be easily degraded, requiring a stable design, and careful epitope selection is required to avoid off-target immunogens, which can lead to autoimmunity. Furthermore, experimental validation of cytokine production and lymphocyte proliferation was not conducted in this study. These assays are planned for future studies to better evaluate the immunogenic potential of the MeFgCatB peptide. Nevertheless, studies investigating the protective efficacy of the MeFgCatB peptide against *F. gigantica* infection are ongoing. To confirm its practical application, future studies will focus on challenge experiments in mice, assessing parasite burden and liver pathology. These studies will help evaluate the protective efficacy of MeFgCatB peptide in vivo.

## 4. Materials and Methods

### 4.1. Sequence Retrieval of Amino Acids

The FgCatB1 (AAO73002), FgCatB2 (AAO73003.1), and FgCatB3 (AAO73004.1) amino acid sequences have been retrieved. The FASTA format of cathepsin B from other species, such as Capra hircus cathepsin B protein (ChCatB, AFC90110), Mus musculus cathepsin B protein (MmCatB, AAA37375), Bos taurus cathepsin B protein (BtCatB, AAA03064), and Homo sapiens cathepsin B protein (HsCatB, AAC37547), was also obtained from the GenBank database (https://www.ncbi.nlm.nih.gov/) accessed on 8 March 2024. Additionally, the Expasy server was used to predict the physicochemical properties of the FgCatB sequence, the AllerTop server version 2.0 was used to predict allergenicity, the Vaxijen server version 2.0 was used to predict antigenicity, and the PepCalc server was used to predict solubility.

### 4.2. Prediction of the BCL Epitopes

The IEDB server with Bepipred Linear Epitope Prediction 2.0 was utilized to predict BCL epitopes based on the epitope/non-epitope predictions for mature FgCatB sequences. An epitope was predicted by scores greater than 0.5 [105]. The Vaxijen version 2.0 and AllerTop version 2.0 were used to assess the antigenicity and allergenicity of the selected epitopes, respectively. Vaxijen relies on protein physicochemical characteristics without recourse to sequence alignment [106]. AllerTop is predicated on a training set that comprises 2427 non-allergens and 2427 recognized allergens from various species [107].

### 4.3. Prediction of the HTL Epitopes

The IEDB server, which we uploaded according to the IEDB recommendations of 2023.05 (NetMHCIIpan 4.1 EL), was used to predict mature FgCatB sequences. The mouse (H-2-I) was the species/locus that was selected, the alleles that were selected were H2-IAb and H2-IAd, and the length that was selected was 15 amino acids. The prediction percentile ranks’ output allows for an accurate assessment of MHC-binding predictions by normalizing the prediction scores for different MHC molecules [108]. The selected epitopes were obtained from the ten percentile rank values. Additionally, Vaxijen version 2.0 and AllerTop version 2.0 were used to predict antigenicity and allergenicity, respectively.

### 4.4. The Novel BCL and HTL Epitopes’ Selection and Construction

The Clustal Omega server version 1.2.4 was used to align the selected BCL and HTL epitopes with the host’s cathepsin B, including HsCatB, BtCatB, MmCatB, and ChCatB. A non-conserved host region, overlapping FgCatB sequences, and the highest percentage conserved residue are among the requirements for the selected BCL and HTL epitopes. Furthermore, the novel BCL and HTL epitopes were constructed using the amino acid sequences of the overlapping epitopes.

### 4.5. Design of the MeFgCatB Peptide

The novel BCL and HTL sequences were used to design the MeFgCatB peptide sequence. The GPGPG (glycine-rich) linker was used to link the novel BCL and HTL sequences. In addition, the percentage identity was used to identify the MeFgCatB peptide with mature sequences from HsCatB, BtCatB, MmCatB, ChCatB, FgCatB1, FgCatB2, and FgCatB3 using the Clustal Omega server version 1.2.4.

### 4.6. Predicting Solubility, Toxicity, Antigenicity, Allergenicity, and Physicochemical Characteristics

The Expasy server was used to predict the physicochemical properties of the MeFgCatB peptide, antigenicity was predicted using the Vaxijen server version 2.0, allergenicity was predicted using the AllerTop server version 2.0, toxicity was predicted using the ToxinPred server version 2.0, and solubility was predicted using the PepCalc server. ToxinPred is based on toxic/non-toxic peptides [109].

### 4.7. Prediction, Refinement, and Validation of the MeFgCatB Peptide Tertiary Structure

The 3D structure of the MeFgCatB peptide was predicted using the AlphaFold2 server. The AlphaFold2 approach is based on predicting the protein structure and structure-based functional annotation [110]. The GalaxyRefine server was used to improve the tertiary model that had the highest local distance difference test (pLDDT) score. The method of GalaxyRefine uses the refined loop and terminus regions using the ab initio modeling [111]. In addition, the MeFgCatB peptide was validated according to the Ramachandran plot and Z-score plot using the SAVES server version 6.0 and the ProSA-web server, respectively.

### 4.8. Predicting Discontinuous and Continuous B-Cell Epitopes

The ElliPro server was used to predict discontinuous and continuous B-cell epitopes. ElliPro is predicated on the 3D structure of a protein antigen, the residue protrusion index (PI), and the nearby residue [112]. The default settings for this server were a minimum score of 0.5 and a maximum distance of 6 Å (Angstrom).

### 4.9. MeFgCatB Peptide with TLR-2 Molecular Docking and Molecular Dynamic (MD) Simulation

The RCSB Protein Data Bank provided the TLR-2 structure (PDB ID: 5D3I). The ClusPro2.0 server was used to dock the vaccination model to TLR-2. The Fast Fourier Transform (FFT) algorithms were used as a basis for the ClusPro2.0 server [113], and a docking complex was selected for analysis. TLR-2 plays a role in enhancing the immune response. Molecular docking was used to study the binding interactions between the peptide vaccine and the TLR-2 receptor. PyMOL 2.5.4 was utilized to visualize the molecular docking. Furthermore, the IMODS server was used for investigating the MD simulation of the docking complex. The PDB file was uploaded to the IMODS server using the selected model, with all parameters maintained at their default settings. Deformability, B-factors, eigenvalues, variance, covariance maps, and the elastic network model were used to assess the stability of the docked model.

### 4.10. In Silico Immune Response Simulation

The immune response simulation was performed using the C-immSim server accessed on 26 March 2024; it can predict immunological interactions using machine learning algorithms and site-specific scoring matrices (PSSM) [58]. Eight hours of real-world time is one time step. The stages in the simulation were selected at 1050. All three doses of the vaccine were boosted at the suggested 28-day interval. The parameters remain the same as the default simulation parameters [86].

### 4.11. The MeFgCatB Peptide Synthesis and Mice Immunization

**Figure 14:**
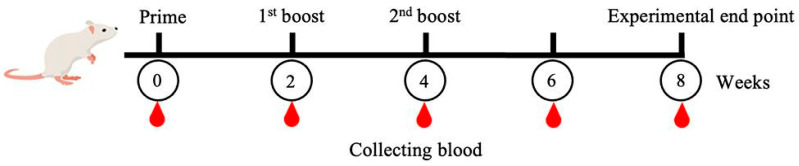
Mice immunization protocol schematic. The MeFgCatB peptide with Quil-A adjuvant was administered to mice three times, two weeks apart (prime, first boost, and second boost). Blood was collected at the time point. All mice were terminated at week 8 for further analysis.

The MeFgCatB peptide sequence was synthesized by GenScript (Singapore) using the PepPower^TM^ peptide synthesis technology platform. The mice experiment was performed using five male, 8-week-old ICR mice. The Animal Care and Use Committee of Burapha University in Thailand approved and managed the experiment according to the procedures (protocol code: IACUC 025/2565; date of approval: 16 September 2022). We obtained blood via the saphenous vein before the vaccination. A total of 50 µg of MeFgCatB was used in the prime dosage for vaccination, and then there were two boosts (first and second boosts) of 25 µg of MeFgCatB peptide each. Individual mice received a subcutaneous injection of the MeFgCatB peptide combined with Quil-A adjuvant (Invitrogen, Carlsbad, CA, USA, Cat. No. 1865007). Three vaccinations were provided to the mice at two-week intervals. The saphenous vein was used for collecting blood from the mice during weeks 0, 2, 4, 6, and 8. At week eight, all mice were terminated (Figure 14).

### 4.12. Parasite Collection

The *F. gigantica* whole-body extracts at different stages, including metacercariae, NEJ, 4WKJ, and adult, were freezes and homogenized with lysis buffer (10 mM Tris–HCl, 10 mM Ethylenediaminetetraacetic acid (EDTA), 150 mM NaCl, and 0.5% Triton X-100, pH 7.4). After mixing the parasite tissues, tissues were centrifuged for 30 min at 4 °C at 12,000× *g*. Following centrifugation, the supernatants were stored at −20 °C. Immunoblotting analysis was performed using the whole-body extracts.

Furthermore, 4% paraformaldehyde was used to fix the fresh NEJ, 4WKJ, and adult stages of *F. gigantica* in PBS (140 mM NaCl, 2.7 mM KCl, 10 mM Na_2_HPO_4_, pH 7.4) at 4 °C for four hrs. A gradient of ethyl alcohol was used to dehydrate the tissues of *F. gigantica* three times for one hour each at concentrations of 70%, 80%, 90%, 95%, and 100%. After that, the *F. gigantica* tissues were cleared with xylene three times, one hour each. Paraplast was infiltrated into the tissues of *F. gigantica* twice for one hour each at 60 °C. The tissues of *F. gigantica* were fixed in paraffin and cut into sections by a microtome that were 5 μm thick. After placing the sections on the 3-aminopropyl-triethoxysilane (Sigma-Aldrich, St. Louis, MO, USA, Cat. No. A3648)-coated slides, they were immediately dried overnight at 40 °C on a hot plate. Immunohistochemistry staining was performed on the parasite sections.

### 4.13. Recombinant Protein Expression

The recombinant *F. gigantica* Cathepsin B1 (rFgCatB1), recombinant *F. gigantica* Cathepsin B2 (rFgCatB2), and recombinant *F. gigantica* Cathepsin B3 (rFgCatB3) were expressed in *E. coli* BL21(DE) and purified as previously described by [38,39]. Briefly, the single colony was picked up and inoculated in 100 mL LB broth, incubated at 37 °C overnight in a shaking incubator. A total of 40 mL of LB broth with 100 µg/mL of kanamycin (Thermo Fisher Scientific, Rockford, IL, USA, Cat. No. 11815024) was added to the incubated culture. This culture was kept in a shaking incubator at 37 °C. When the culture reached an OD600 of 0.6, 1 mM Isopropyl β-D-thiogalactopyranoside (IPTG) (Sigma-Aldrich, St. Louis, MO, USA, Cat. No. I5502) was added. Two hours were utilized for incubating this culture. The culture was subsequently centrifuged for 30 min at 4000× *g*. Pellets were collected. The pellets were used to perform the protein purification by Ni-NTA affinity chromatography (QIAGEN, Germantown, MD, USA, Cat. No. 30210). The rFgCatB1, rFgCatB2, and rFgCatB3 were eluted by denaturing conditions. The SnakeSkin^TM^ Pleated Dialysis Tubing (Thermo Scientific, Rockford, IL, USA, Cat. No. 68110) was used to dialyze the elutes. Protein concentrations were measured using the Amicon Ultra centrifugal filter devices (Millipore, Bedford, MA, USA, Cat. No. UFC500308). Lowry’s technique was used to determine the protein concentrations [114].

### 4.14. Determination of IgG1 and IgG2a Levels by Indirect ELISA

Indirect ELISA was used to measure the levels of IgG1 and IgG2a in mouse serum in triplicate. A total of 50 μL of 2 μg/mL of the MeFgCatB synthesis peptide in coating buffer (35 mM NaHCO_3_ and 15 mM Na_2_CO_3_, pH 9.6) was added to a U96 Maxi Sorp Nunc-Immuno Plate (Thermo Fisher Scientific, Waltham, MA, USA, Cat. No. 446261) and kept overnight at 4 °C. Washing with 0.05% PBST was performed on the coated plates three times. A total of 100 μL of 1% bovine serum albumin (BSA) (Capricorn Science, Ebsdorfergrund, Germany, Cat. No. BSA-1000) was added to each well to prevent nonspecific binding, and the wells were then incubated for one hour at room temperature (RT). Mouse sera diluted in PBS were added to the plates at 1:50 after washing three times with 0.05% PBST, and the plates were then incubated for two hours at RT. Following incubation, plates were rinsed three times with 0.05% PBST and then incubated for two hours at RT with horseradish peroxidase (HRP)-conjugated goat anti-mouse IgG1 or IgG2a (Southern Biotech, Birmingham, AL, USA, Cat. No. 5300-05) diluted with PBS at a ratio of 1:500. After five washes with 0.05% PBST, 50 μL of 3, 3′, 5, 5′-tetramethylbenzidine (TMB) (Sigma, St. Louis, MO, USA, Cat. No. T0440) was added to the plates, and they were incubated for 15 min at RT. For the stop reaction, 50 μL of stop buffer (1 N HCl) was added to each well. An automatic SpectraMax ABS Microplate Reader (Molecular Devices, San Jose, CA, USA) was used to measure optical densities (OD) at 450 nm. Furthermore, IgG1 and IgG2a levels for the proteins rFgCatB1, rFgCatB2, and rFgCatB3 were measured at week 8 according with the previous method.

### 4.15. Immunoblotting Analysis

The *F. gigantica* whole-body extracts at different stages, including metacercariae, NEJ, 4WKJ, and adult stages, were separated by using 12.5% SDS-PAGE and electrically transferred to Amersham™ Protran**^®^** Western blotting membranes (Cytiva, Marlborough, MA, USA, Cat. No. 10600002). The membranes were subsequently nonspecifically blocked for one hour at RT using 4% BSA in PBS. Mice serum was diluted in PBS and added to the membranes, which were then incubated for one hour at RT. Following incubation, the membranes were washed three times with 0.1% PBST for five minutes each. Alkaline phosphatase (AP)-conjugated goat anti-mouse IgG (Invitrogen, Carlsbad, CA, USA, Cat. No. 31320) was subsequently added and diluted with PBS. The membranes were then incubated for one hour at RT. The membranes were washed three times with 0.1% PBST for five minutes. After that, the membranes were incubated with alkaline phosphatase (AP) buffer (0.1 M NaCl and 0.1 M Tris-HCl, pH 9.5) for five minutes at RT. The color in a positive band was developed by the nitroblue tetrazolium chloride/5-bromo-4-chloro-3-indodyl phosphate (NBT/BCIP) substrates (Merck, Darmstadt, Germany, Cat. No. 11681451001). The membranes were stopped from reacting by stop buffer (TBS, 20 mM EDTA, pH 8.0).

### 4.16. Localization of the F. gigantica Tissue

The parasite slides were dewaxed using xylene and rehydrated through a graded ethanol series (100%, 95%, 80%, and 70%) for five minutes each. Antigen retrieval was performed by microwaving the sections three times for five minutes at 700 W in 10 mM citrate buffer (pH 6.0). After rinsing with tap water for five minutes, the parasite slides were added with 0.1% PBST and blocked with 4% BSA in PBS for one hour to prevent nonspecific binding. The parasite slides were then incubated overnight at 4 °C with MeFgCatB peptide-immunized sera diluted in PBS containing 1% BSA. After washing with 0.1% PBST, the parasite slides were incubated for one hour at RT with AP-conjugated goat anti-mouse IgG. The color was developed using NBT/BCIP substrates in the dark, and the reaction was stopped using stop buffer. Finally, the slides were observed under a light microscope.

## 5. Conclusions

This study utilized an immunoinformatic technique to design the MeFgCatB peptide, which was highly antigenic, non-toxic, non-allergic, and had good solubility. The synthesized MeFgCatB peptide can induce IgG1 and IgG2a, indicating a mixed type of Th1/Th2 immune response with Th2 predominating. The MeFgCatB peptide-immunized sera were able to detect the rFgCatB1, rFgCatB2, and rFgCatB3 proteins. The MeFgCatB peptide-immunized sera were specific to all stages of the *F. gigantica*. In addition, it can detect caecal epithelium in the NEJ, 4WKJ, and adult stages of the *F. gigantica*. However, the percent protection provided by the MeFgCatB peptide should be investigated further in future experiments.

## Figures and Tables

**Table 1:** Properties of *F. gigantica* cathepsin Bs.

Proteins	Accession ID	Antigenicity	Allergen	Solubility	MW (kDa)	GRAVY
FgCatB1	AAO73002	0.4738	Yes	Good	27.977	−0.419
FgCatB2	AAO73003	0.5909	No	Poor	28.842	−0.559
FgCatB3	AAO73004	0.4678	No	Good	27.625	−0.465

FgCatB1, *F. gigantica* cathepsin B1; FgCatB2, *F. gigantica* cathepsin B2; FgCatB3, *F. gigantica* cathepsin B3; kDa, kilodaltons; MW, molecular weight; GRAVY, the grand average of hydropathicity.

**Table 2:** Selected BCL epitopes predicted from FgCatB sequences.

No.	Start	End	Peptide Sequences	Lengths (aa)	Antigenicity	Allergenicity
**FgCatB1**						
1	47	54	NGEKKPRL	8	0.9741	No
2	66	72	CGYGCEG	7	3.3484	No
3	221	227	NEGWGEN	7	1.4453	No
**FgCatB2**						
1	47	54	NGQMRPRL	8	1.7296	No
2	66	72	CGQGCRG	7	2.0161	No
**FgCatB3**						
1	47	54	NGQKKPRL	8	0.8798	No
2	82	144	WTREGVVTGGTLENPTGCLPYPFPKCSHGVVTPGLPPCPRDIYPTPKCEKKCHAGYNKTYEQD	63	0.7660	No

FgCatB1, *F. gigantica* cathepsin B1; FgCatB2, *F. gigantica* cathepsin B2; FgCatB3, *F. gigantica* cathepsin B3; aa, amino acid.

**Table 3:** HTL epitopes for the FgCatB sequences.

No.	Allele	Start	End	Peptide Sequence	Antigenicity	Allergenicity
**FgCatB1**						
1	H2-IAb	80	94	DYWWRHGIVSGGTLE	1.2868	No
2	H2-IAb	82	96	WWRHGIVSGGTLENP	1.6390	No
3	H2-IAb	83	97	WRHGIVSGGTLENPT	1.4469	No
4	H2-IAb	84	98	RHGIVSGGTLENPTG	1.0879	No
5	H2-IAb	123	137	LYATPKCEKQCQAGY	0.9071	No
6	H2-IAb	184	198	YKSGIYQYTSGSLMG	0.5161	No
7	H2-IAb	187	201	GIYQYTSGSLMGGHG	0.6313	No
**FgCatB2**						
1	H2-IAb	28	42	SCWATAAASAMSDRV	0.7423	No
2	H2-IAb	84	98	REGIVTGGTWENRTG	1.6318	No
3	H2-IAb	116	130	YSRCPHYTYPTPPCA	0.7205	No
4	H2-IAb	117	131	SRCPHYTYPTPPCAR	0.9486	No
5	H2-IAb	118	132	RCPHYTYPTPPCARA	0.9528	No
6	H2-IAb	142	156	EQDKFYGNSSYNVGE	0.7000	No
7	H2-IAb	144	158	DKFYGNSSYNVGEHE	0.6180	No
**FgCatB3**						
1	H2-IAb	84	98	REGVVTGGTLENPTG	1.4574	No
2	H2-IAb	107	121	CSHGVVTPGLPPCPR	1.0781	No
3	H2-IAb	108	122	SHGVVTPGLPPCPRD	1.1865	No
4	H2-IAb	192	206	TTGRLVGGHAIRVIG	0.6327	No
5	H2-IAb	193	207	TGRLVGGHAIRVIGW	0.9272	No

FgCatB1, *F. gigantica* cathepsin B1; FgCatB2, *F. gigantica* cathepsin B2; FgCatB3, *F. gigantica* cathepsin B3.

**Table 4:** The percentage of conserved residues of the overlapped BCL and HTL groups.

Group	Conserved Residues (aa)	Total Residue (aa)	Percentage of Conserved Residues
**BCL epitope**			
B1	5	8	62.50
**HTL epitope**			
T1	11	15	73.33

B1, BCL 1 epitope groups; T1, HTL 1 epitope groups; aa, amino acids.

**Table 5:** The residue numbers of discontinuous and continuous B-cell epitopes.

No.	Residues	Residue Numbers
**Discontinuous epitopes**		
1	A:N25, A:P26, A: T27, A:G28	4
2	A:N1, A:G2, A:Q3, A:K4	4
**Continuous epitopes**		
1	A:L23, A:E24, A:N25, A:P26, A:T27, A:G28	6
2	A:N1, A:G2, A:Q3, A:K4, A:K5, A:P6, A:R7, A:L8	8

## Data Availability

The datasets generated or analyzed during this study are available from the corresponding author on reasonable request.

## References

[1] Nyindo M., Lukambagire A.H. (2015). Fascioliasis: An Ongoing Zoonotic Trematode Infection. BioMed Res. Int..

[2] Mostafa W., Abdel-Rady A., El-Dakroury M.F., Felefel W. (2023). Field Trials to Evaluate Five Fasciolicides against Natural Liver Fluke Infection in Cattle and Sheep in Egypt. Int. J. Vet. Sci..

[3] Tolan R.W. (2011). Fascioliasis Due to *Fasciola hepatica* and *Fasciola gigantica* Infection: An Update on This “neglected” Neglected Tropical Disease. Lab. Med..

[4] Rosas-Hostos Infantes L.R., Paredes Yataco G.A., Ortiz-Martínez Y., Mayer T., Terashima A., Franco-Paredes C., Gonzalez-Diaz E., Rodriguez-Morales A.J., Bonilla-Aldana D.K., Vargas Barahona L. (2023). The Global Prevalence of Human Fascioliasis: A Systematic Review and Meta-Analysis. Ther. Adv. Infect. Dis..

[5] Winaya I.B.O., Oka I.B.M., Adnyana I.B.W., Sudipa P.H. (2023). Fibrosis and Collagen-I Accumulation in Bali Cattle Liver Tissue Infected with *Fasciola gigantica*. Int. J. Vet. Sci..

[6] WHO (2007). Report of the WHO Informal Meeting on Use of Triclabendazole in Fascioliasis Control.

[7] Fairweather I. (2005). Triclabendazole: New Skills to Unravel an Old(Ish) Enigma. J. Helminthol..

[8] Cwiklinski K., Jewhurst H., McVeigh P., Barbour T., Maule A.G., Tort J., O’Neill S.M., Robinson M.W., Donnelly S., Dalton J.P. (2018). Infection by the Helminth Parasite Fasciola Hepatica Requires Rapid Regulation of Metabolic, Virulence, and Invasive Factors to Adjust to Its Mammalian Host. Mol. Cell. Proteom..

[9] Beesley N.J., Cwiklinski K., Allen K., Hoyle R.C., Spithill T.W., James La Course E., Williams D.J.L., Paterson S., Hodgkinson J.E. (2023). A Major Locus Confers Triclabendazole Resistance in Fasciola Hepatica and Shows Dominant Inheritance. PLoS Pathog..

[10] Martínez-Moreno A., Jiménez V., Martínez-Cruz M.S., Martínez-Moreno F.J., Becerra C., Hernández S. (1997). Triclabendazole Treatment in Experimental Goat Fasciolosis: Anthelmintic Efficacy and Influence in Antibody Response and Pathophysiology of the Disease. Vet. Parasitol..

[11] Kelley J.M., Elliott T.P., Beddoe T., Anderson G., Skuce P., Spithill T.W. (2016). Current Threat of Triclabendazole Resistance in Fasciola Hepatica. Trends. Parasitol..

[12] Cabada M.M., Lopez M., Cruz M., Delgado J.R., Hill V., White A.C. (2016). Treatment Failure after Multiple Courses of Triclabendazole among Patients with Fascioliasis in Cusco, Peru: A Case Series. PLoS Negl. Trop. Dis..

[13] Morales M.L., Tanabe M.B., White A.C., Lopez M., Bascope R., Cabada M.M. (2021). Triclabendazole Treatment Failure for Fasciola Hepatica Infection among Preschool and School-Age Children, Cusco, Peru. Emerg. Infect. Dis..

[14] Dalton J.P., Neill S.O., Stack C., Collins P., Walshe A., Sekiya M., Doyle S., Mulcahy G., Hoyle D., Khaznadji E. (2003). Fasciola Hepatica Cathepsin L-like Proteases: Biology, Function, and Potential in the Development of First Generation Liver Fluke Vaccines. Int. J. Parasitology.

[15] Flores-Velázquez L.M., Ruiz-Campillo M.T., Herrera-Torres G., Martínez-Moreno Á., Martínez-Moreno F.J., Zafra R., Buffoni L., Rufino-Moya P.J., Molina-Hernández V., Pérez J. (2023). Fasciolosis: Pathogenesis, Host-Parasite Interactions, and Implication in Vaccine Development. Front. Vet. Sci..

[16] ur Rehman T., Elsaid F.G., Toledo M.M.G., Gentile A., Gul R.A., Rashid M., Aleem M.T., Zaman M.A. (2023). Fasciolosis: Recent Update in Vaccines Development and Their Efficacy. Pak. Vet. J..

[17] Robinson M.W., Dalton J.P. (2009). Zoonotic Helminth Infections with Particular Emphasis on Fasciolosis and Other Trematodiases. Philos. Trans. R. Soc. B Bio. Sci..

[18] Sangpairoj K., Apisawetakan S., Changklungmoa N., Kueakhai P., Chaichanasak P., Sobhon P., Chaithirayanon K. (2018). Potential of Recombinant 2-Cys Peroxiredoxin Protein as a Vaccine for *Fasciola gigantica* Infection. Exp. Parasitol..

[19] Dalton J.P., McGonigle S., Rolph T.P., Andrews S.J. (1996). Induction of Protective Immunity in Cattle against Infection with *Fasciola hepatica* by Vaccination with Cathepsin L Proteinases and with Hemoglobin. Infect. Immun..

[20] Kueakhai P., Changklungmoa N., Waseewiwat P., Thanasinpaiboon T., Cheukamud W., Chaichanasak P., Sobhon P. (2017). Characterization and Vaccine Potential of *Fasciola gigantica* Saposin-like Protein 1 (SAP-1). Vet. Parasitol..

[21] Kueakhai P., Changklungmoa N., Riengrojpitak S., Chaichanasak P., Meemon K., Chaithirayanon K., Chantree P., Sansri V., Itagaki T., Sobhon P. (2013). Vaccine Potential of Recombinant Saposin-like Protein 2 against *Fasciolosis gigantica* in Mice. Vaccine.

[22] Jaikua W., Kueakhai P., Chaithirayanon K., Tanomrat R., Wongwairot S., Riengrojpitak S., Sobhon P., Changklungmoa N. (2016). Cytosolic Superoxide Dismutase Can Provide Protection against *Fasciola gigantica*. Acta Trop..

[23] Preyavichyapugdee N., Sahaphong S., Riengrojpitak S., Grams R., Viyanant V., Sobhon P. (2008). *Fasciola gigantica* and *Schistosoma mansoni*: Vaccine Potential of Recombinant Glutathione S-Transferase (RFgGST26) against Infections in Mice. Exp. Parasitol..

[24] Kumar N., Anju V., Gaurav N., Chandra D., Samanta S., Gupta S.C., Adeppa J., Raina O.K. (2012). Vaccination of Buffaloes with *Fasciola gigantica* Recombinant Glutathione S-Transferase and Fatty Acid Binding Protein. Parasitol. Res..

[25] López-Abán J., Esteban A., Vicente B., Rojas-Caraballo J., del Olmo E., Martínez-Fernández A.R., Hillyer G.V., Muro A. (2012). Adaptive Immune Stimulation Is Required to Obtain High Protection with Fatty Acid Binding Protein Vaccine Candidate against *Fasciola hepatica* in Balb/C Mice. J. Parasitol..

[26] Changklungmoa N., Kueakhai P., Riengrojpitak S., Chaithirayanon K., Chaichanasak P., Preyavichyapugdee N., Chantree P., Sansri V., Itagaki T., Sobhon P. (2013). Immunization with Recombinant Leucine Aminopeptidase Showed Protection against *Fasciola gigantica* in Mice. Parasitol. Res..

[27] Raina O.K., Nagar G., Varghese A., Prajitha G., Alex A., Maharana B.R., Joshi P. (2011). Lack of Protective Efficacy in Buffaloes Vaccinated with *Fasciola gigantica* Leucine Aminopeptidase and Peroxiredoxin Recombinant Proteins. Acta Trop..

[28] Piacenza L., Acosta D., Basmadjian I., Dalton J.P., Carmona C. (1999). Vaccination with Cathepsin L Proteinases and with Leucine Aminopeptidase Induces High Levels of Protection against Fascioliasis in Sheep. Infect. Immun..

[29] Golden O., Flynn R.J., Read C., Sekiya M., Donnelly S.M., Stack C., Dalton J.P., Mulcahy G. (2010). Protection of Cattle against a Natural Infection of Fasciola Hepatica by Vaccination with Recombinant Cathepsin L1 (RFhCL1). Vaccine.

[30] Villa-Mancera A., Reynoso-Palomar A., Utrera-Quintana F., Carreón-Luna L. (2014). Cathepsin L1 Mimotopes with Adjuvant Quil A Induces a Th1/Th2 Immune Response and Confers Significant Protection against *Fasciola hepatica* Infection in Goats. Parasitol. Res..

[31] Sansri V., Meemon K., Changklungmoa N., Kueakhai P., Chantree P., Chaichanasak P., Lorsuwannarat N., Itagaki T., Sobhon P. (2015). Protection against *Fasciola gigantica* Infection in Mice by Vaccination with Recombinant Juvenile-Specific Cathepsin L.. Vaccine.

[32] Kueakhai P., Changklungmoa N., Cheukamud W., Osotprasit S., Chantree P., Preyavichyapugdee N., Sobhon P., Meemon K. (2021). The Combined Recombinant Cathepsin L1H and Cathepsin B3 Vaccine against *Fasciola gigantica* Infection. Parasitol. Int..

[33] Changklungmoa N., Phoinok N., Yencham C., Sobhon P., Kueakhai P. (2016). Vaccine Potential of Recombinant CathepsinL1G against *Fasciola gigantica* in Mice. Vet. Parasitol..

[34] Kueakhai P., Changklungmoa N., Chaichanasak P., Jaikua W., Itagaki T., Sobhon P. (2015). Vaccine Potential of Recombinant Pro- and Mature CathepsinL1 against *Fasciolosis gigantica* in Mice. Acta Trop..

[35] Chantree P., Phatsara M., Meemon K., Chaichanasak P., Changklungmoa N., Kueakhai P., Lorsuwannarat N., Sangpairoj K., Songkoomkrong S., Wanichanon C. (2013). Vaccine Potential of Recombinant Cathepsin B against *Fasciola gigantica*. Exp. Parasitol..

[36] Tort J., Brindley P.J., Knox D., Wolfe K.H., Dalton J.P. (1999). Proteinases and Associated Genes of Parasitic Helminths. Adv. Parasitol..

[37] Meemon K., Grams R., Vichasri-Grams S., Hofmann A., Korge G., Viyanant V., Upatham E.S., Habe S., Sobhon P. (2004). Molecular Cloning and Analysis of Stage and Tissue-Specific Expression of Cathepsin B Encoding Genes from *Fasciola gigantica*. Mol. Biochem. Parasitol..

[38] Sethadavit M., Meemon K., Jardim A., Spithill T.W., Sobhon P. (2009). Identification, Expression and Immunolocalization of Cathepsin B3, a Stage-Specific Antigen Expressed by Juvenile *Fasciola gigantica*. Acta Trop..

[39] Chantree P., Wanichanon C., Phatsara M., Meemon K., Sobhon P. (2012). Characterization and Expression of Cathepsin B2 in *Fasciola gigantica*. Exp. Parasitol..

[40] Montaner S., Galiano A., Trelis M., Martin-Jaular L., del Portillo H.A., Bernal D., Marcilla A. (2014). The Role of Extracellular Vesicles in Modulating the Host Immune Response during Parasitic Infections. Front. Immunol..

[41] Capron A., Dessaint J.P., Haque A., Capron M. (1982). Antibody-Dependent Cell-Mediated Cytotoxicity against Parasites. Prog. Allergy.

[42] Rezaei M., Nazari M. (2022). New Generation Vaccines for COVID-19 Based on Peptide, Viral Vector, Artificial Antigen Presenting Cell, DNA or MRNA. Avicenna J. Med. Biotechnol..

[43] Malonis R.J., Lai J.R., Vergnolle O. (2020). Peptide-Based Vaccines: Current Progress and Future Challenges. Chem. Rev..

[44] Skwarczynski M., Toth I. (2016). Peptide-Based Synthetic Vaccines. Chem. Sci..

[45] Bartlett B.L., Pellicane A.J., Tyring S.K. (2009). Vaccine Immunology. Dermatol. Ther..

[46] Rastogi I., Jeon D., Moseman J.E., Muralidhar A., Potluri H.K., McNeel D.G. (2022). Role of B Cells as Antigen Presenting Cells. Front. Immunol..

[47] Shawan M.M.A.K., Sharma A.R., Halder S.K., Al Arian T., Shuvo M.N., Sarker S.R., Hasan M.A. (2023). Advances in Computational and Bioinformatics Tools and Databases for Designing and Developing a Multi-Epitope-Based Peptide Vaccine. Int. J. Pept. Res. Ther..

[48] de Melo T.T., Mendes M.M., Alves C.C., Carvalho G.B., Fernandes V.C., Pimenta D.L.F., de Moraes Mourão M., Gai F., Kalli M., Coelho A. (2019). The *Schistosoma mansoni* Cyclophilin A Epitope 107-121 Induces a Protective Immune Response against Schistosomiasis. Mol. Immunol..

[49] Gazzinelli-Guimarães A.C., Nogueira D.S., Amorim C.C.O., Oliveira F.M.S., Coqueiro-Dos-Santos A., Carvalho S.A.P., Kraemer L., Barbosa F.S., Fraga V.G., Santos F.V. (2021). ASCVac-1, a Multi-Peptide Chimeric Vaccine, Protects Mice Against *Ascaris suum* Infection. Front. Immunol..

[50] Zawawi A., Forman R., Smith H., Mair I., Jibril M., Albaqshi M.H., Brass A., Derrick J.P., Else K.J. (2020). In Silico Design of a T-Cell Epitope Vaccine Candidate for Parasitic Helminth Infection. PLoS Pathog..

[51] Gu Y., Sun X., Huang J., Zhan B., Zhu X. (2020). A Multiple Antigen Peptide Vaccine Containing CD4^+^ T Cell Epitopes Enhances Humoral Immunity against *Trichinella spiralis* Infection in Mice. J. Immunol. Res..

[52] Chansap S., Cheukamud W., Suthisintong T., Kueakhai P., Changklungmoa N. (2025). Development of Multi-Epitope Cathepsin L Driven Short Peptide Vaccine against *Fasciola gigantica*. Front. Vet. Sci..

[53] Rojas-Caraballo J., López-Abán J., Pérez Del Villar L., Vizcaíno C., Vicente B., Fernández-Soto P., Del Olmo E., Patarroyo M.A., Muro A. (2014). In Vitro and in Vivo Studies for Assessing the Immune Response and Protection-Inducing Ability Conferred by Fasciola Hepatica-Derived Synthetic Peptides Containing B- and T-Cell Epitopes. PLoS ONE.

[54] Vakili B., Nezafat N., Zare B., Erfani N., Akbari M., Ghasemi Y., Rahbar M.R., Hatam G.R. (2020). A New Multi-Epitope Peptide Vaccine Induces Immune Responses and Protection against *Leishmania infantum* in BALB/c Mice. Med. Microbiol. Immunol..

[55] Guo L., Yang H., Tang F., Yin R., Liu H., Gong X., Wei J., Zhang Y., Xu G., Liu K. (2017). Oral Immunization with a Multivalent Epitope-Based Vaccine, Based on NAP, Urease, HSP60, and HpaA, Provides Therapeutic Effect on *H. pylori* Infection in Mongolian Gerbils. Front. Cell. Infect. Microbiol..

[56] Zhang B.Z., Wang X., Yuan S., Li W., Dou Y., Poon V.K.M., Chan C.C.S., Cai J.P., Chik K.K.H., Tang K. (2021). A Novel Linker-Immunodominant Site (LIS) Vaccine Targeting the SARS-CoV-2 Spike Protein Protects against Severe COVID-19 in *Syrian hamsters*. Emerg. Microbes Infect..

[57] Lei R., Kim W., Lv H., Mou Z., Scherm M.J., Schmitz A.J., Turner J.S., Tan T.J.C., Wang Y., Ouyang W.O. (2023). Leveraging Vaccination-Induced Protective Antibodies to Define Conserved Epitopes on Influenza N2 Neuraminidase. Immunity.

[58] Rapin N., Lund O., Bernaschi M., Castiglione F. (2010). Computational Immunology Meets Bioinformatics: The Use of Prediction Tools for Molecular Binding in the Simulation of the Immune System. PLoS ONE.

[59] Plotkin S., Robinson J.M., Cunningham G., Iqbal R., Larsen S. (2017). The Complexity and Cost of Vaccine Manufacturing—An Overview. Vaccine.

[60] Raoufi E., Hemmati M., Eftekhari S., Khaksaran K., Mahmodi Z., Farajollahi M.M., Mohsenzadegan M. (2020). Epitope Prediction by Novel Immunoinformatics Approach: A State-of-the-Art Review. Int. J. Pept. Res. Ther..

[61] Hamley I.W. (2022). Peptides for Vaccine Development. ACS Appl. Bio. Mater..

[62] Ge H., Sun L., Yu J. (2017). Fast Batch Searching for Protein Homology Based on Compression and Clustering. BMC Bioinform..

[63] Vojtek I., Buchy P., Doherty T.M., Hoet B. (2019). Would Immunization Be the Same without Cross-Reactivity?. Vaccine.

[64] Trier N.H., Houen G. (2023). Antibody Cross-Reactivity in Auto-Immune Diseases. Int. J. Mol. Sci..

[65] Schaap-Johansen A.L., Vujović M., Borch A., Hadrup S.R., Marcatili P. (2021). T Cell Epitope Prediction and Its Application to Immunotherapy. Front. Immunol..

[66] Reddy Chichili V.P., Kumar V., Sivaraman J. (2013). Linkers in the Structural Biology of Protein-Protein Interactions. Protein Sci..

[67] Khan M., Khan S., Ali A., Akbar H., Sayaf A.M., Khan A., Wei D.Q. (2019). Immunoinformatics Approaches to Explore Helicobacter Pylori Proteome (Virulence Factors) to Design B and T Cell Multi-Epitope Subunit Vaccine. Sci. Rep..

[68] Yurina V., Adianingsih O.R. (2022). Predicting Epitopes for Vaccine Development Using Bioinformatics Tools. Ther. Adv. Vaccines Immunother..

[69] Chen X., Zaro J.L., Shen W.C. (2013). Fusion Protein Linkers: Property, Design and Functionality. Adv. Drug Deliv. Rev..

[70] Ayyagari V.S., Venkateswarulu T.C., Abraham Peele K., Srirama K. (2022). Design of a Multi-Epitope-Based Vaccine Targeting M-Protein of SARS-CoV2: An Immunoinformatics Approach. J. Biomol. Struct. Dyn..

[71] Segal Y., Shoenfeld Y. (2018). Vaccine-Induced Autoimmunity: The Role of Molecular Mimicry and Immune Crossreaction. Cell. Mol. Immunol..

[72] Gamage D.G., Gunaratne A., Periyannan G.R., Russell T.G. (2019). Applicability of Instability Index for In Vitro Protein Stability Prediction. Protein Pept. Lett..

[73] Chaplin D.D. (2010). Overview of the Immune Response. J. Allergy Clin. Immunol..

[74] Wiltgen M. (2018). Algorithms for Structure Comparison and Analysis: Homology Modelling of Proteins. Encyclopedia of Bioinformatics and Computational Biology: ABC of Bioinformatics.

[75] Wiederstein M., Sippl M.J. (2007). ProSA-Web: Interactive Web Service for the Recognition of Errors in Three-Dimensional Structures of Proteins. Nucleic Acids Res..

[76] Park S.W., Lee B.H., Song S.H., Kim M.K. (2023). Revisiting the Ramachandran Plot Based on Statistical Analysis of Static and Dynamic Characteristics of Protein Structures. J. Struct. Biol..

[77] Ferdous S., Kelm S., Baker T.S., Shi J., Martin A.C.R. (2019). B-Cell Epitopes: Discontinuity and Conformational Analysis. Mol. Immunol..

[78] Khanmohammadi S., Rezaei N. (2021). Role of Toll-like Receptors in the Pathogenesis of COVID-19. J. Med. Virol..

[79] Kalita P., Lyngdoh D.L., Padhi A.K., Shukla H., Tripathi T. (2019). Development of Multi-Epitope Driven Subunit Vaccine against *Fasciola gigantica* Using Immunoinformatics Approach. Int. J. Biol. Macromol..

[80] Basto A.P., Leitão A. (2014). Targeting TLR2 for Vaccine Development. J. Immunol. Res..

[81] Oliveira-Nascimento L., Massari P., Wetzler L.M. (2012). The Role of TLR2 Ininfection and Immunity. Front. Immunol..

[82] Das K.C., Konhar R., Biswal D.K. (2023). *Fasciola gigantica* Vaccine Construct: An in Silico Approach towards Identification and Design of a Multi-Epitope Subunit Vaccine Using Calcium Binding EF-Hand Proteins. BMC Immunol..

[83] Kalita J., Padhi A.K., Tripathi T. (2020). Designing a Vaccine for Fascioliasis Using Immunogenic 24 KDa Mu-Class Glutathione s-Transferase. Infect. Genet. Evol..

[84] Akıl M., Aykur M., Karakavuk M., Can H., Döşkaya M. (2022). Construction of a Multiepitope Vaccine Candidate against Fasciola Hepatica: An in Silico Design Using Various Immunogenic Excretory/Secretory Antigens. Expert Rev. Vaccines.

[85] López-Blanco J.R., Aliaga J.I., Quintana-Ortí E.S., Chacón P. (2014). IMODS: Internal Coordinates Normal Mode Analysis Server. Nucleic Acids Res..

[86] Sanches R.C.O., Tiwari S., Ferreira L.C.G., Oliveira F.M., Lopes M.D., Passos M.J.F., Maia E.H.B., Taranto A.G., Kato R., Azevedo V.A.C. (2021). Immunoinformatics Design of Multi-Epitope Peptide-Based Vaccine Against *Schistosoma mansoni* Using Transmembrane Proteins as a Target. Front. Immunol..

[87] Chatterjee D., Al Rimon R., Chowdhury U.F., Islam M.R. (2023). A Multi-Epitope Based Vaccine against the Surface Proteins Expressed in Cyst and Trophozoite Stages of Parasite *Entamoeba histolytica*. J. Immunol. Methods.

[88] Jyotisha, Qureshi R., Qureshi I.A. (2023). Development of a Multi-Epitope Vaccine Candidate for Leishmanial Parasites Applying Immunoinformatics and in Vitro Approaches. Front. Immunol..

[89] Saha S., Vashishtha S., Kundu B., Ghosh M. (2022). In-Silico Design of an Immunoinformatics Based Multi-Epitope Vaccine against *Leishmania donovani*. BMC Bioinform..

[90] Sarfraz A., Wara T.U., Sheheryar, Chen K., Ansari S.H., Zaman A., Nishan U., Iqbal A., Ullah R., Ali E.A. (2023). Structural Informatics Approach for Designing an Epitope-Based Vaccine against the Brain-Eating *Naegleria fowleri*. Front. Immunol..

[91] Aziz S., Waqas M., Halim S.A., Ali A., Iqbal A., Iqbal M., Khan A., Al-Harrasi A. (2022). Exploring Whole Proteome to Contrive Multi-Epitope-Based Vaccine for NeoCoV: An Immunoinformtics and in-Silico Approach. Front. Immunol..

[92] Dalton J.P., Robinson M.W., Mulcahy G., O’Neill S.M., Donnelly S. (2013). Immunomodulatory Molecules of *Fasciola hepatica*: Candidates for Both Vaccine and Immunotherapeutic Development. Vet. Parasitol..

[93] Meira C.d.S., Gedamu L. (2019). Protective or Detrimental? Understanding the Role of Host Immunity in Leishmaniasis. Microorganisms.

[94] Liang S., Zhang S., Bao Y., Zhang Y., Liu X., Yao H., Liu G. (2024). Combined Immunoinformatics to Design and Evaluate a Multi-Epitope Vaccine Candidate against *Streptococcus suis* Infection. Vaccines.

[95] Umar A., Haque A., Alghamdi Y.S., Mashraqi M.M., Rehman A., Shahid F., Khurshid M., Ashfaq U.A. (2021). Development of a Candidate Multi-Epitope Subunit Vaccine against *Klebsiella aerogenes*: Subtractive Proteomics and Immuno-Informatics Approach. Vaccines.

[96] Petrovsky N., Aguilar J.C. (2004). Vaccine Adjuvants: Current State and Future Trends. Immunol. Cell Biol..

[97] Singh M., O’Hagan D.T. (2003). Recent Advances in Veterinary Vaccine Adjuvants. Int. J. Parasitol..

[98] Reed S.G., Orr M.T., Fox C.B. (2013). Key Roles of Adjuvants in Modern Vaccines. Nat. Med..

[99] Bian L., Zheng Y., Guo X., Li D., Zhou J., Jing L., Chen Y., Lu J., Zhang K., Jiang C. (2022). Intramuscular Inoculation of AS02-Adjuvanted Respiratory Syncytial Virus (RSV) F Subunit Vaccine Shows Better Efficiency and Safety Than Subcutaneous Inoculation in BALB/c Mice. Front. Immunol..

[100] Abdelallah N.H., Abdeltawab N.F., Boseila A.A., Amin M.A. (2016). Chitosan and Sodium Alginate Combinations Are Alternative, Efficient, and Safe Natural Adjuvant Systems for Hepatitis B Vaccine in Mouse Model. Evid.-Based Complement. Altern. Med..

[101] Rivera F., Espino A.M. (2016). Adjuvant-Enhanced Antibody and Cellular Responses to Inclusion Bodies Expressing FhSAP2 Correlates with Protection of Mice to *Fasciola hepatica*. Exp. Parasitol..

[102] Mielke D., Bandawe G., Pollara J., Abrahams M.R., Nyanhete T., Moore P.L., Thebus R., Yates N.L., Kappes J.C., Ochsenbauer C. (2019). Antibody-Dependent Cellular Cytotoxicity (ADCC)-Mediating Antibodies Constrain Neutralizing Antibody Escape Pathway. Front. Immunol..

[103] Arts E.J., Hazuda D.J. (2012). HIV-1 Antiretroviral Drug Therapy. Cold Spring Harb. Perspect. Med..

[104] Groß A., Hashimoto C., Sticht H., Eichler J. (2016). Synthetic Peptides as Protein Mimics. Front. Bioeng. Biotechnol..

[105] Jespersen M.C., Peters B., Nielsen M., Marcatili P. (2017). BepiPred-2.0: Improving Sequence-Based B-Cell Epitope Prediction Using Conformational Epitopes. Nucleic Acids Res..

[106] Doytchinova I.A., Flower D.R. (2007). VaxiJen: A Server for Prediction of Protective Antigens, Tumour Antigens and Subunit Vaccines. BMC Bioinform..

[107] Dimitrov I., Naneva L., Doytchinova I., Bangov I. (2014). AllergenFP: Allergenicity Prediction by Descriptor Fingerprints. Bioinformatics.

[108] Reynisson B., Alvarez B., Paul S., Peters B., Nielsen M. (2021). NetMHCpan-4.1 and NetMHCIIpan-4.0: Improved Predictions of MHC Antigen Presentation by Concurrent Motif Deconvolution and Integration of MS MHC Eluted Ligand Data. Nucleic Acids Res..

[109] Gupta S., Kapoor P., Chaudhary K., Gautam A., Kumar R., Raghava G.P.S. (2015). Peptide Toxicity Prediction. Methods Mol. Biol..

[110] Bryant P., Pozzati G., Elofsson A. (2022). Improved Prediction of Protein-Protein Interactions Using AlphaFold2. Nat. Commun..

[111] Heo L., Park H., Seok C. (2013). GalaxyRefine: Protein Structure Refinement Driven by Side-Chain Repacking. Nucleic Acids Res..

[112] Ponomarenko J., Bui H.H., Li W., Fusseder N., Bourne P.E., Sette A., Peters B. (2008). ElliPro: A New Structure-Based Tool for the Prediction of Antibody Epitopes. BMC Bioinform..

[113] Vajda S., Yueh C., Beglov D., Bohnuud T., Mottarella S.E., Xia B., Hall D.R., Kozakov D. (2017). New Additions to the ClusPro Server Motivated by CAPRI. Proteins Struct. Funct. Bioinform..

[114] Lowry O.H., Rosebrough N.J., Farr A.L., Randall R.J. (1951). Protein Measurement with the Folin Phenol Reagent. J. Biol. Chem..

